# Low molecular weight species of TDP-43 generated by abnormal splicing form inclusions in amyotrophic lateral sclerosis and result in motor neuron death

**DOI:** 10.1007/s00401-015-1412-5

**Published:** 2015-03-19

**Authors:** Shangxi Xiao, Teresa Sanelli, Helen Chiang, Yulong Sun, Avijit Chakrabartty, Julia Keith, Ekaterina Rogaeva, Lorne Zinman, Janice Robertson

**Affiliations:** 1 Tanz Centre for Research in Neurodegenerative Diseases, University of Toronto, Toronto, ON M5T 2S8 Canada; 2 Department of Laboratory Medicine and Pathobiology, University of Toronto, Toronto, ON M5T 2S8 Canada; 3 Department of Medical Biophysics, Ontario Cancer Institute, University of Toronto, TMDT 4-305, 101 College Street, Toronto, ON M5G 1L7 Canada; 4 Sunnybrook Health Sciences Centre, Toronto, ON M4N 3M5 Canada

**Keywords:** Amyotrophic lateral sclerosis, Frontotemporal lobar degeneration, TDP-43, TDP-35, C-terminal species, Alternative splicing, Alternative translation

## Abstract

The presence of lower molecular weight species comprising the C-terminal region of TAR DNA-binding protein 43 (TDP-43) is a characteristic of TDP-43 proteinopathy in amyotrophic lateral sclerosis (ALS) and frontotemporal lobar degeneration (FTLD). Here, we have identified a novel splice variant of TDP-43 that is upregulated in ALS and generates a 35-kDa N-terminally truncated species through use of an alternate translation initiation codon (ATG^Met85^), denoted here as Met^85^-TDP-35. Met^85^-TDP-35 expressed ectopically in human neuroblastoma cells exhibited reduced solubility, cytoplasmic distribution, and aggregation. Furthermore, Met^85^-TDP-35 sequestered full-length TDP-43 from the nucleus to form cytoplasmic aggregates. Expression of Met^85^-TDP-35 in primary motor neurons resulted in the formation of Met^85^-TDP-35-positive cytoplasmic aggregates and motor neuron death. A neo-epitope antibody specific for Met^85^-TDP-35 labeled the 35-kDa lower molecular weight species on immunoblots of urea-soluble extracts from ALS-FTLD disease-affected tissues and co-labeled TDP-43-positive inclusions in ALS spinal cord sections, confirming the physiological relevance of this species. These results show that the 35-kDa low molecular weight species in ALS-FTLD can be generated from an abnormal splicing event and use of a downstream initiation codon and may represent a mechanism by which TDP-43 elicits its pathogenicity.

**Electronic supplementary material:**

The online version of this article (doi:10.1007/s00401-015-1412-5) contains supplementary material, which is available to authorized users.

## Introduction

TAR DNA-binding protein of 43 kDa (TDP-43) is a nuclear DNA/RNA binding protein that has numerous functions in regulating RNA metabolism, including transcription, alternative splicing, miRNA processing and mRNA stabilization [3]. TDP-43 proteinopathy is characterized by the formation of neuronal cytoplasmic inclusions containing TDP-43, and this pathology is common to the majority of amyotrophic lateral sclerosis (ALS) as well as frontotemporal lobar degeneration (FTLD) cases [1, 16]. Biochemically, pathological TDP-43 comprises the normal full-length protein; an abnormally phosphorylated form that runs at 45 kDa; and lower molecular weight species of 25–35 kDa that correspond to N-terminally truncated forms of TDP-43. Expression of lower molecular weight species in cell culture recapitulates features of disease, including formation of cytoplasmic aggregates and recruitment of full-length TDP-43 from the nucleus [9, 24, 26, 29]. For C-terminal species of ~35 kDa, these effects led to decreased cell viability, impaired neurite outgrowth in neuronal cell lines, and a loss of TDP-43 splicing activity [9, 24, 26, 29]. These results suggest that C-terminal species of TDP-43 can drive pathogenesis and as such understanding their etiology is important for therapy development. In this regard, the 35-kDa species described above were generated from different starting amino acids. Prior studies have shown that species of 35 and 25 kDa can be generated by caspase-3 cleavage at DETD^89^ and DVMD^219^, respectively, a phenomenon that could be triggered upon suppression of progranulin expression in cell culture [31]. Similarly, another study found that induction of apoptosis in cultured cells with staurosporine also led to the activation of caspase-3 and the subsequent generation of 25-kDa and 35-kDa TDP-43 fragments [11]. However, species of 35 and 25 kDa are also found in cells lacking caspase-3 [17]. As such, although the TDP-43 caspase-3 cleavage product runs at the same molecular weight as the 35-kDa C-terminal species observed in disease tissues on SDS–polyacrylamide gel electrophoresis, one cannot infer with absolute certainty that the 35-kDa species in disease tissues is indeed derived from caspase-3 cleavage. Therefore, given the role of TDP-43 in splicing of other genes [4, 6, 7, 14] and the fact that it is alternatively spliced itself [25], we explored alternative splicing and/or translation as a potential mechanism for generating pathological TDP-43 C-terminal species in ALS. To this end, we have identified an alternatively spliced variant of TDP-43 that is upregulated in ALS and has 91 bp spliced out in exon 2, generating a protein species of 35 kDa through use of a downstream start codon, ATG^Met85^, denoted here as Met^85^-TDP-35. Use of ATG^Met85^ as a TDP-43 start codon has previously been shown in cell culture studies, however, the physiological relevance of this was unknown [17]. Here, using a neo-epitope antibody specifically recognizing Met^85^-TDP-35, we have shown that the pathologically associated TDP-43 species of 35 kDa in ALS-FTLD can be generated through use of ATG^Met85^. The Met^85^-TDP-35 neo-epitope antibody not only labeled the 35-kDa species on immunoblots of disease tissues, but also co-labeled TDP-43-positive inclusions in ALS spinal cord tissue, showing that Met^85^-TDP-35 is fully integrated within the pathology associated with the disease. These findings indicate that pathological variants of TDP-43 can be generated through a combination of alternative splicing and translation, mechanisms that could be targeted therapeutically.

## Materials and methods

### ALS and control cases

Spinal cord tissues were collected from eleven sALS cases, four with *C9orf72* repeat expansions, and one fALS case also carrying a *C9orf72* repeat expansion [27]; and one case each of Alzheimer’s disease, corticobasal degeneration, multisystem atrophy and Huntington’s disease (Table 1). Prefrontal cortex tissues were collected from six ALS cases with pathologically confirmed FTLD, and six neurologically normal control cases (Table 1). ALS cases were diagnosed using the Revised El Escorial Criteria (Brooks 2000), and patient consents were obtained from the legal representatives in accordance with the Ethical Review Boards of Sunnybrook Health Sciences Centre and University of Toronto (Toronto, Canada).

**Table 1:** ALS and control cases used in this study

Cases	Sex	Age (years)	Diagnosis	Gene mutation
ALS cases				
A1	F	49	sALS-FTLD	*C9orf72*
A2	F	46	sALS	N/A
A3	F	73	sALS	N/A
A4	M	70	sALS	*C9orf72*
A5	F	58	sALS-FTLD	*C9orf72*
A6	F	59	sALS	N/A
A7	M	52	sALS	N/A
A8	F	65	sALS	N/A
A9	M	58	fALS-FTLD	*C9orf72*
A10	F	60	sALS	N/A
A11	M	64	sALS-FTLD	*C9orf72*
A12	F	57	sALS	N/A
A13	F	83	sALS-FTLD	N/A
A14	F	71	sALS-FTLD	N/A
Control cases				
C1	F	91	AD	N/A
C2	M	77	CBD	N/A
C3	M	69	MSA	N/A
C4	F	78	HD	N/A
C5	M	76	N.N.	N/A
C6	M	74	N.N.	N/A
C7	M	89	N.N.	N/A
C8	F	48	N.N.	N/A
C9	M	54	N.N.	N/A
C10	F	82	N.N.	N/A

*sALS* sporadic ALS, *fALS* familial ALS, *FTLD* frontotemporal lobar degeneration, *C9orf72* chromosome 9 open reading frame 72, *N/A* not applicable, *AD* Alzheimer’s disease, *CBD* corticobasal degeneration, *MSA* multiple system atrophy, *HD* Huntington’s disease, *N.N.* neurologically normal

### RT-PCR and cloning of TDP-43 and its splice variant

Fifty milligrams of gray matter was dissected from the anterior horn of postmortem lumbar spinal cord from twelve ALS and four control cases, from which total RNA was extracted using TRIzol Reagent (Life Technologies) according to the manufacturer’s protocol and treated with DNase to minimize DNA contamination. The RNA transcripts were first normalized with β-actin primers (Life Technologies) after synthesizing complementary DNA (cDNA) from 1 μg of total RNA using random hexamers and the SuperScript III First-strand Synthesis System for RT-PCR (Life Technologies) following manufacturer’s protocol. PCR was performed with 2 μl of template cDNA mixed with 18 μl of reaction mix containing 10 μl of 2× Master Mix (Life Technologies), 6 μl water, 1 μl of 10 μM forward primer (5′-ATG TCT GAA TAT ATT CGG GTA AC-3′) and 1 μl of 10 μM reverse primer (5′-GTC CAT CTA TCA TAT GTC GCT GTG-3′). Amplification of TDP-43 and its splice variant was performed in a 9800 Fast Thermal Cycler (Life Technologies) with an initial denaturation at 95 °C for 10 s, followed by 35 cycles of denaturation at 95 °C for 0 s and annealing at 66 °C for 15 s. The amplified products were visualized on a 1.5 % (w/v) agarose/ethidium bromide gels and subjected to sequence verification.

For cloning of full-length TDP-43 and its splice variant, 1 μg total RNA isolated from ALS spinal cords was reverse transcribed with an adapter-tagged oligo dT primer (oligo(dT)17-GGC CAC GCG TCG ACT AGT AC) into cDNA, which was purified with a Rapid Amplification of cDNA Ends (RACE) Purification System (Life Technologies). Poly-(dC) sequences were added to the N-terminus of 10 μl cDNA using a TdT-tailing reaction (6.5 μl DEPC-treated water, 5 μl of 5× tailing buffer, 2.5 μl of 2 mM dCTP, and 1 μl TdT) according to manufacturer’s protocol (Life Technologies). PCR was performed with 5 μl of tailed cDNA with 45 μl PCR reaction mix containing 1× PfuUltra buffer, 200 μM dNTP, 0.2 μM 5′RACE abridged anchor primer (5′-GGC CAC GCG TCG ACT AGT ACG GGI IGG GII GGG IIG-3′), 0.2 μM adapter primer (5′-GGC CAC GCG TCG ACT AGT AC-3′), and 1.25 U PfuUltra DNA polymerase (Agilent Technologies). Amplification of PCR products was performed with an initial denaturation at 95 °C for 45 s, followed by 25 cycles of denaturation at 95 °C for 45 s, 55 °C for 45 s, and 68 °C for 90 s in GeneAmp PCR System 9700 (Life Technologies). The amplified products were purified with Qiagen PCR Purification Kit (Qiagen) and were subjected to a second round of amplification with a 5′UTR forward primer with a NotI restriction site (5′-ataagaatgcggccgcGTC CGT CGC TGC TTC GGT G-3′) and a 3′UTR reverse primer with a BamHI restriction site (5′-atacgcggatccGCC TGT GAT GCG TGA TGA CG-3′). The PCR conditions were the same as the first round except with 30 cycles. The amplified products were visualized on a 1.0 % (w/v) agarose/ethidium bromide gel, purified with the MiniElute Gel Extraction Kit (Qiagen), and then subcloned into the NotI/BamHI restriction sites of the mammalian expression vector pcDNA3.1(−). The Met^85^-TDP-35 construct, starting at the 3rd ATG (Met85) of full-length TDP-43, was generated by PCR and cloned into pcDNA3.1(−) expression vector using a forward primer containing a BamHI restriction site (5′-gcggatccaccATG GAT GAG ACA GAT GCT TC-3′) and a reverse primer containing a KpnI restriction site (5′-cacggtacCTA CAT TCC CCA GCC AGA AGA CTT AG-3′). For visualization of the subcellular localization of full-length TDP-43 and Met^85^-TDP-35, these two sequences were cloned into pEGFP-C2 and pmRFP-C2 vectors, for N-terminal tagging with EGFP and RFP, respectively. Sequence analysis was performed on all constructs to ensure correct sequences.

### Silver staining

PCR amplified products were resolved on 10 % native PAGE (polyacrylamide gel electrophoresis) gels followed by fixation in 10 % ethanol, 0.05 % acetic acid for 30 min. Gels were then stained with a 10 mM silver nitrate solution for 30 min, developed in a 350 mM sodium hydroxide, 0.3 % formaldehyde solution, and the reaction stopped using 5 % acetic acid. Gel images were captured and band intensities quantified using Image J software with values expressed as mean ± standard error of the mean (SEM).

### Cell culture and microinjection of primary motor neurons

The human neuroblastoma cell line SH-SY5Y was maintained in DMEM/F12 medium (Life Technologies) supplemented with 10 % fetal bovine serum (FBS). Transfection was performed with the corresponding plasmid DNAs using Lipofectamine 2000 (Life Technologies) following manufacturer’s protocol. Generation of primary motor neuron cultures was as described previously [21, 28], and plasmids containing EGFP-tagged full-length TDP-43 and EGFP-tagged Met^85^-TDP-35 in pEGFP-C2 vector were microinjected at 200 ng/μl into motor neuron nuclei along with 20 μg/μl of the fluorescent marker Dextran FITC (Life Technologies). Cell viability was determined daily by counting the number of FITC-positive motor neurons, which was normalized to the number of FITC-positive motor neurons on day 1 after microinjection. All experiments were performed in triplicate and values were expressed as mean ± SEM.

### Immunocytochemistry of cultured cells

To visualize EGFP-tagged construct expression, cultured cells grown on glass coverslips were fixed with 3.7 % (w/v) paraformaldehyde in phosphate-buffered saline (PBS) for 20 min at room temperature (RT) followed by 3 × 5 min PBS washes then counterstained with 4′,6-diamidino-2-phenylindole (DAPI) nucleic acid stain for 1 min (Life Technologies). Immunofluorescence was visualized using a Leica DMI6000 microscope and images captured using Volocity software (PerkinElmer).

### Protein extraction and Western blotting

SH-SY5Y cells grown on 35 mm^2^ dishes (Thermo Scientific) were harvested in 1 ml PBS containing protease inhibitors at 4 °C, and the cell suspensions were centrifuged at 500×*g* for 5 min at 4 °C. The resulting pellets were homogenized in 1 ml low salt buffer [50 mM Tris, pH 7.5, 150 mM NaCl, 5 mM ethylenediaminetetraacetic acid (EDTA)] containing protease inhibitors (Sigma-Aldrich) and then centrifuged at 16,100×*g* for 20 min at 4 °C. Supernatants were saved as the low salt fraction, and pellets were homogenized in a high salt Triton X-100 buffer (50 mM Tris, pH 7.5, 750 mM NaCl, 5 mM EDTA, 1 % Triton X-100, and protease inhibitors), followed by centrifugation at 16,100×*g* for 20 min at 4 °C. Supernatants were saved as the high salt Triton X-100 fraction, and the remaining pellets were solubilized in urea buffer (7 M urea, 2 M thiourea, 4 % 3-[(3-cholamidopropyl)dimethylammonio]-1-propanesulfonate (CHAPS), 30 mM Tris–HCl, pH 8.5) and saved as the urea fraction.

For human tissues, gray matter from ALS and control lumbar spinal cord was dissected and weighed, then homogenized at 200 mg/ml using the same biochemical fractionation procedure as for cultured cells but with one exception: after extraction in high salt Triton X-100 buffer, the resulting pellets were homogenized in a high salt sucrose buffer (50 mM Tris, pH 7.5, 750 mM NaCl, 5 mM EDTA, 30 % sucrose, and protease inhibitors), followed by centrifugation at 16,100×*g* for 30 min at 4 °C to float and remove myelin. For immunoblotting, sodium dodecyl sulfate (SDS) sample buffer (10 mM Tris–HCl, pH 6.8, 1 mM EDTA, 40 mM DTT, 1 % SDS, and 10 % sucrose) was added to 50 μg of each sample fraction, which was heated at 95 °C for 5 min with the exception of the urea fraction to avoid carbamoylation of proteins. Equivalent amounts of protein samples were loaded onto 10 % SDS-PAGE gels and transferred to polyvinyldiflouride (PVDF) membranes, which were blocked in 5 % skim milk dissolved in Tris-buffered saline (TBS) containing 0.2 % Tween-20 for 1 h at RT, followed by overnight incubation at 4 °C with a polyclonal TDP-43 antibody (1:1000, Proteintech), a polyclonal N-terminal TDP-43 antibody (1:1000, Aviva Systems Biology), or a polyclonal C-terminal TDP-43 antibody (1:1000, generated in-house using a synthetic peptide, FGSSMDSKSSGWG, linked to keyhole limpet hemocyanin as the antigen). Bound antibody was visualized with an ECL Detection System (PerkinElmer) using HRP-conjugated secondary antibodies.

To extract insoluble proteins from the prefrontal cortex, six ALS cases with pathologically confirmed FTLD-TDP-43, and six neurological normal controls of a similar age group were included. Tissue was dissected from Brodmann’s area 10, weighed, and homogenized at 250 mg/ml in low salt buffer (10 mM Tris–HCl, pH 7.5, 5 mM EDTA, 1 mM DTT, 10 % sucrose, and protease inhibitors) with a glass Dounce homogenizer (Sigma-Aldrich). Homogenate was centrifuged at 25,000×*g* for 30 min at 4 °C, and the resulting supernatant was saved as the low salt fraction. Pellet was homogenized at 250 mg/ml (starting tissue weight) in high salt buffer (low salt buffer + 500 mM NaCl, 1 % Triton X-100, and protease inhibitors) and centrifuged at 180,000×*g* for 30 min at 4 °C, and the resulting supernatant was saved as the high salt fraction. Pellet was homogenized in 250 mg/ml (starting tissue weight) myelin flotation buffer (10 mM Tris–HCl, pH 7.5, 500 mM NaCl, 5 mM EDTA, 1 mM DTT, 40 % sucrose, 1 % Triton X-100, and protease inhibitors) and centrifuged at 180,000×*g* for 30 min at 4 °C, and the floating myelin removed. The resulting supernatant was saved as the myelin flotation fraction. Pellet was homogenized in 50 mg/ml (starting tissue weight) sarkosyl buffer (low salt buffer + 500 mM NaCl, 1 % *N*-lauroyl-sarcosine, and protease inhibitors) and incubated with agitation at 22 °C for 30 min, followed by centrifugation at 180,000×*g* for 30 min at 22 °C. The resulting supernatant was saved as the sarkosyl fraction. Pellet was sonicated with the Ultrasonic Cell Disrupter (Misonix) in 1 g/ml (starting tissue weight) urea buffer (30 mM Tris–HCl, pH 8.5, 7 M urea, 2 M thiourea, 4 % CHAPS) and saved as the urea fraction. All fractions were resuspended with SDS sample buffer and all samples except for the urea fraction were heated at 95 °C for 5 min prior to SDS-PAGE and immunoblot analyses.

### Generation of neo-epitope antibodies

Rabbit polyclonal antibodies specifically recognizing Met^85^-TDP-35 or caspase-3-cleaved TDP-35 (C3-TDP-35) were generated using synthetic peptides corresponding to the neo-epitopes generated by alternative translation initiation at ATG^Met85^ (M^85^DETDASSA) or caspase-3 cleavage at DETD^89^ (A^90^SSAVKVKR), respectively, linked to keyhole limpet hemocyanin (ProSci Inc.). Both antibodies were diluted 1:1000 for immunoblotting and 1:500 for immunohistochemistry.

### Generation of recombinant TDP-43 protein

Full-length TDP-43 was expressed as a fusion protein with His-tagged Venus YFP at the N-terminus joined by a TEV-cleavable linker. The construct was cloned into the pET-30 vector (EMD Millipore) and expressed in *E. coli* BL21-AI cells (Life Technologies). Bacterial culture was lysed by sonication on ice with lysis buffer (40 mM HEPES–KOH, pH 7.4, 500 mM KCl, 20 mM MgCl_2_, 10 % glycerol, 20 mM imidazole, 2 mM β-mercaptoethanol) containing protease inhibitors (Roche Applied Science). Lysate was centrifuged at 15,000 RPM in a Sorvall SS-34 rotor for 30 min at 4 °C. Prior to Ni–NTA agarose bead column (Qiagen) purification of the lysate supernatant, the column was washed with 5 column volumes of 40 mM HEPES–KOH, pH 7.4, 500 mM KCl, 20 mM MgCl_2_, 10 % glycerol, 20 mM imidazole, 2 mM β-mercaptoethanol, and protease inhibitors. The YFP-TDP-43 fusion protein was eluted from the column with 40 mM HEPES, pH 7.4, 500 mM KCl, 20 mM MgCl_2_, 250 mM imidazole, 10 % glycerol, 2 mM β-mercaptoethanol, and protease inhibitors. SDS-PAGE analysis of eluent showed that the fusion protein fraction was 95 % pure.

### In vitro caspase-3 cleavage of recombinant TDP-43

To generate caspase-3-cleaved TDP-43, 2 μg of recombinant human YFP-tagged TDP-43 was incubated with 2 U of active human recombinant caspase-3 (EMD Millipore) in a reaction mix containing 100 mM NaCl, 50 mM HEPES, 10 mM DTT, 1 mM EDTA, 10 % glycerol, and 0.1 % CHAPS, pH 7.4, at 37 °C for 4 h. The reaction was halted by adding 2× SDS sample buffer.

### Immunohistochemistry of human spinal cord tissues

Formalin-fixed paraffin-embedded lumbar spinal cord tissues from ALS and control cases were cut into 4 μm serial sections and then mounted on glass slides. Tissue sections were rehydrated in sequential washes of xylene, xylene:ethanol (1:1), and decreasing concentrations of ethanol, followed by a final wash in PBS. Blocking of endogenous peroxidase activity was achieved by incubation with 3 % (v/v) hydrogen peroxide, and antigens retrieved by incubation in either 10 mM citrate buffer at pH 6.0 for polyclonal TDP-43 antibody (ProteinTech) or Tris–EDTA buffer (20 mM Tris–HCl, 0.65 mM EDTA, 0.0005 % Tween-20, pH 9.0, for Met^85^-TDP-35 antibody). Sections were incubated overnight at RT with polyclonal TDP-43 antibody (1:5000, Proteintech) or Met^85^-TDP-35 antibody (1:500) diluted in antibody diluent (0.05 M Tris–HCl, pH 7.4, 0.25 % Tween-20, 0.2 % Triton X-100, 1 % casein, and 0.05 % thimerosol). Bound antibody was visualized using the ImmPRESS Anti-rabbit Ig Peroxidase Kit (Vector Labs) for polyclonal TDP-43 antibody or with the Super Sensitive Polymer HRP Kit (Biogenex) for Met^85^-TDP-35 antibody labeling. Color development was achieved using NovaRed (Vector Labs) and sections were counterstained with hematoxylin. Images were captured using a QImaging Micropublisher 3.3 RTV digital camera attached to a Leica DM6000 upright microscope and Openlab Imaging software (PerkinElmer).

For double immunofluorescence labeling, formalin-fixed paraffin-embedded spinal cords were cut into 6 μm sections and subjected to the same processing as above, except that the primary antibodies were rabbit polyclonal Met^85^-TDP-35 antibody (1:500) and mouse monoclonal TDP-43 antibody (2E2-D3, 1:500, Novus Biologicals) and the secondary antibodies were goat anti-rabbit Alexa Fluor 488 and goat anti-mouse Alexa Fluor 594, respectively. Images were captured with an ORCA ER Hamamatsu digital camera mounted on a Leica DMI600 inverted microscope using Volocity Imaging software (PerkinElmer).

## Results

### Identification of a novel splice variant of TDP-43

**Fig. 1:**
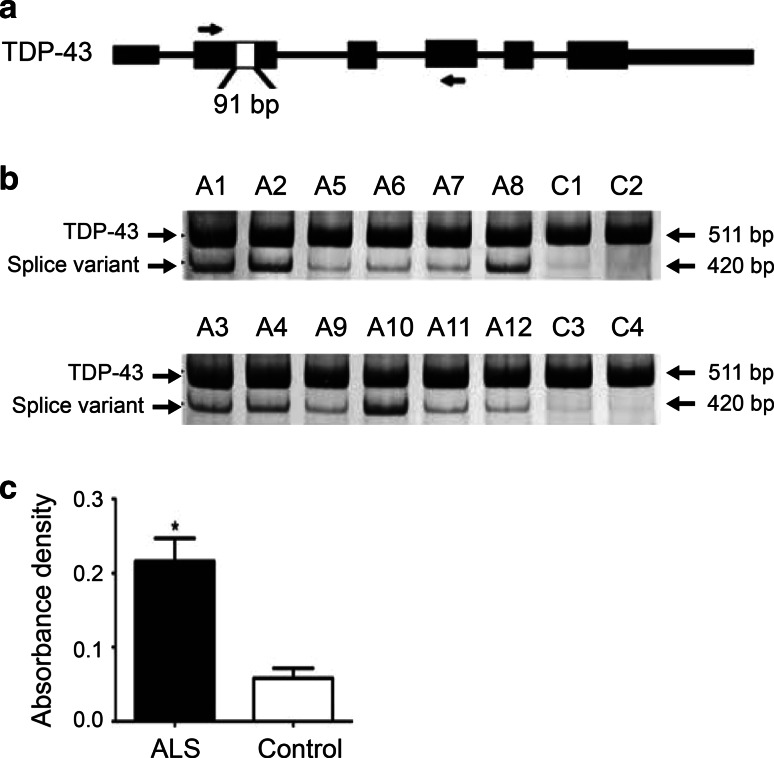
TDP-43 r.[106_196del] splice variant is upregulated in ALS. **a** *TARDBP* gene structure showing splicing deletion. *Black boxes* are exons. *White box* in exon 2 denotes the 91 bp skipped by alternative splicing. *Arrows* indicate positions of the forward and reverse primers for RT-PCR. **b** RT-PCR amplification of the TDP-43 r.[106_196del] splice variant using RNA isolated from ALS and control spinal cord. Region amplified has 511 bp in the constitutively spliced transcript and 420 bp in the alternatively spliced transcript. Cases *A1*, *A4*, *A5*, *A9*, and *A11* also carry *C9orf72* expansions. **c** Quantification using ImageJ showed a ~4 fold upregulation of the splice variant in ALS (*n* = 12; 0.216 ± 0.031) compared to controls (*n* = 4; 0.059 ± 0.013), *p* < 0.0126

A search of EST sequences in Genbank (http://www.ncbi.nlm.nih.gov/genbank/) yielded two human (accession numbers: BI464234 and BG435185) and fourteen mouse (accession numbers: AV506877, AW 106495, BU610138, BY740492, BY788083, CA319629, CA543520, CA879844, CD553499, CD766458, CN691221, CN692254, CN696163, and DV060848) EST sequences, which revealed the same 91 bp splicing deletion in exon 2 of *TARDBP*, encoding TDP-43. This splice variant uses a non-canonical splicing pair (UU:AG), thus skipping base pairs 106–196 within exon 2 (denoted here as TDP-43 r.[106_196del]). To investigate the relevance of this splice variant in ALS, we selected twelve cases (11 sALS and one fALS with *C9orf72* expansions), all of which exhibited TDP-43- and ubiquitin-positive inclusions upon postmortem examination, and 4 control cases for transcript analysis. RT-PCR was performed using total RNA isolated from lumbar spinal cords, with a forward primer located in exon 2 and a reverse primer in exon 4 of *TARDBP* (Fig. 1a). A major band of 511 bp, corresponding to the full-length TDP-43 transcript, was present across all ALS and control cases. A lower band of 420 bp that corresponds to the alternatively spliced variant TDP-43 r.[106_196del] was found at much higher levels in ALS cases compared to controls (Fig. 1b). After normalization of total RNA to β-actin, quantification analysis showed that the TDP-43 r.[106_196del] transcript was upregulated by ~4.0 fold (*p* < 0.0126) in ALS (*n* = 12; 0.216 ± 0.031) compared to controls (*n* = 4; 0.059 ± 0.013) (Fig. 1c).

### TDP-35 is an N-terminally truncated protein with reduced solubility

**Fig. 2:**
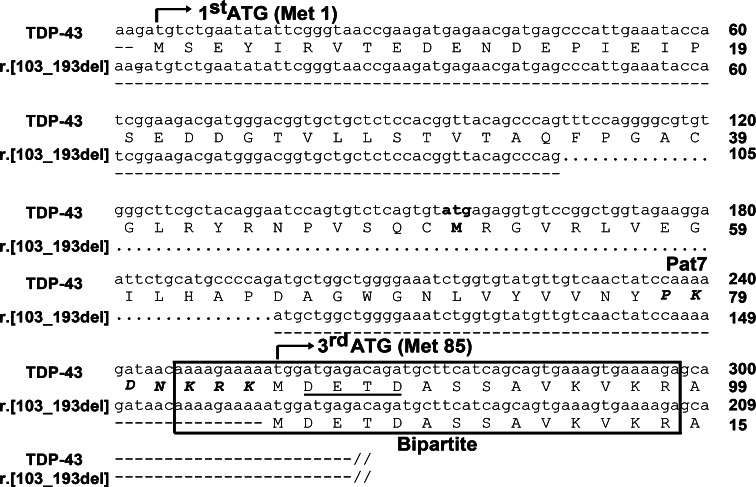
Sequence alignment of TDP-43 and the TDP-43 r.[106_196del] splice variant. The 91 bp splicing deletion is signified by *dotted lines*. Pat7 (aa 78–84, shown in *bold italics*) and bipartite (aa 82–98, *boxed*) nuclear localization sequences are indicated. Note that ATG^Met85^ immediately precedes the caspase-3 cleavage site DETD^89^ (*underlined*)

Translation tool analysis (http://www.expasy.org/tools/) of the TDP-43 splice variant transcript showed that if it was transcribed from the first ATG^Met1^, the 91 bp deletion in exon 2 would cause a frame shift and generate a premature stop codon (TAG) 190–192 bp downstream from ATG^Met1^, thereby producing a short protein of 63 aa with a pI/MW of 4.32/7.2 kDa. Theoretically, such mRNA transcripts containing premature stop codons would be eliminated through nonsense-mediated mRNA decay [8], which was corroborated in our findings as we did not detect a protein product of this size across our studies. However, translation analysis also revealed the presence of a complete open reading frame (ORF) situated 161 bp downstream from ATG^Met1^. The ATG initiation codon of this ORF corresponds to the third ATG^Met85^ of full-length TDP-43, with the loss of the second ATG^Met51^ through the splicing deletion (Fig. 2).

**Fig. 3:**
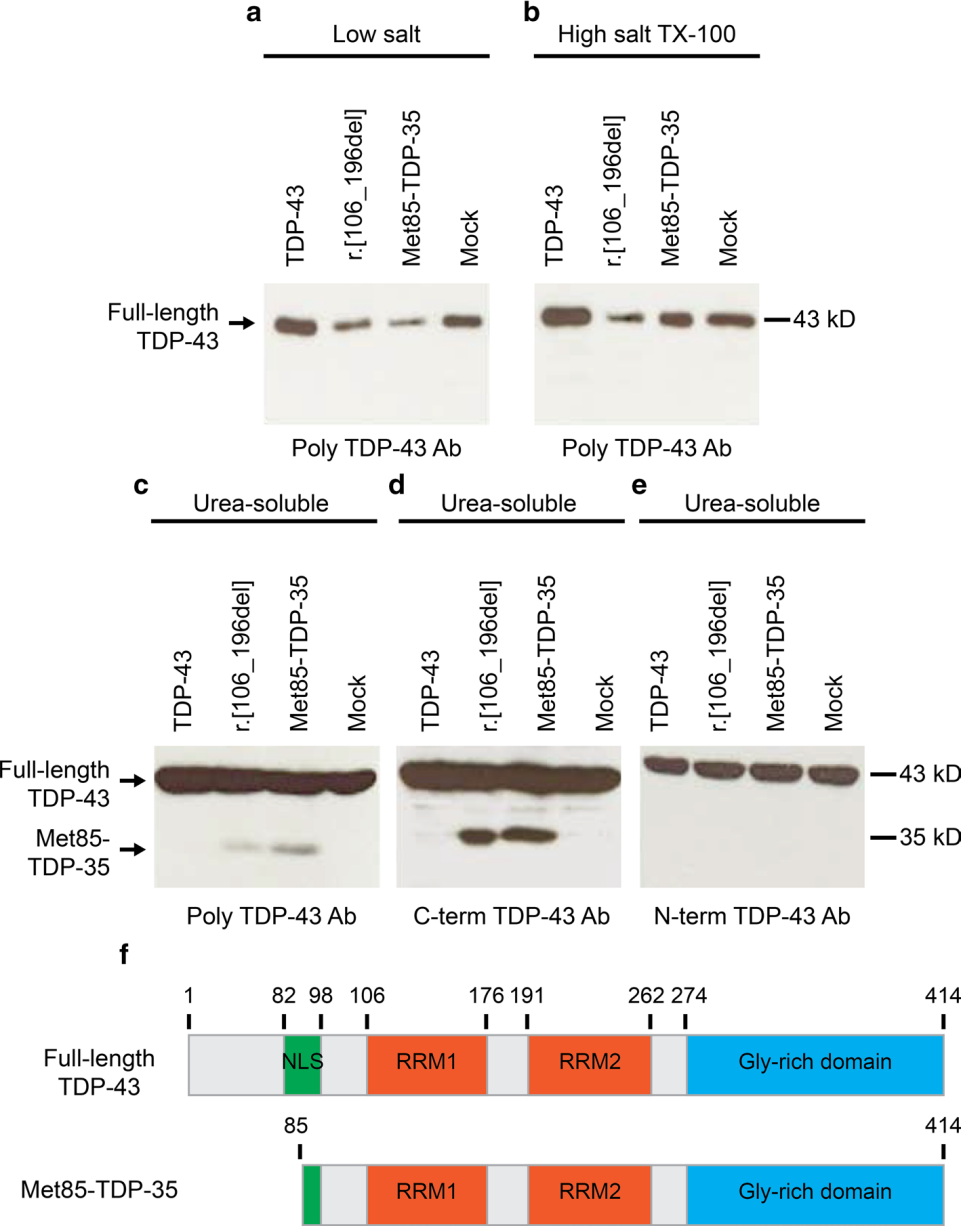
TDP-43 r.[106_196del] splice variant generates an N-terminally truncated protein of 35 kDa. SH-SY5Y cells expressing full-length TDP-43, TDP-43 r.[106_196del], Met^85^-TDP-35, or mock vector were fractionated in buffers of increasing stringency: **a** low salt; **b** high salt-TX-100; **c**–**e** urea buffer. Full-length TDP-43 was found in all fractions on immunoblots probed with polyclonal TDP-43. A lower molecular weight species of 35 kDa was observed only in urea-soluble fractions of lysates from cells expressing TDP-43 r.[106_196del] or Met^85^-TDP-35 (**c**) and was detected using antibody to the C-terminus of TDP-43 (**d**) but not to the N-terminus (**e**). **f** Comparative structures of TDP-43 and Met^85^-TDP-35

Expression of the TDP-43 r.[106_196del] cDNA in pcDNA3.1(−) in human neuroblastoma (SH-SY5Y) cells resulted in the generation of a 35-kDa protein product on immunoblots of cell lysates, thereby confirming the prediction described above (Fig. 3). To biochemically characterize this 35-kDa species, SH-SY5Y cells expressing full-length TDP-43, TDP-43 r.[106_196del], or Met^85^-TDP-35 (generated by PCR) were harvested and proteins sequentially extracted with buffers of increasing stringency, followed by immunoblot analysis of all fractions (Fig. 3). Full-length TDP-43 distributed to all fractions, as has been reported previously, whereas the 35-kDa species expressed from TDP-43 r.[106_196] and Met^85^-TDP-35 was exclusively partitioned to the urea-soluble fraction (Fig. 3), indicating a decreased solubility compared to the full-length protein. Both TDP-43 r.[106_196del] and Met^85^-TDP-35 were detected with an antibody to the C-terminus of TDP-43, but not to the N-terminus, confirming the loss of the N-terminal domain in these proteins (Fig. 3).

### TDP-35 has disrupted nuclear localization signals and forms cytoplasmic aggregates in human neuroblastoma cells

Given that Met^85^-TDP-35 is translated from the third ATG^Met85^ of full-length TDP-43, there is disruption of two NLS sequences located within the N-terminal region of the full-length protein, namely, the Pat7 NLS from aa 78–84 (PKDNKRK) and the bipartite NLS from aa 82–98 (KRKMDETDASSAVKVKR) (Fig. 2). To examine the subcellular localization of full-length TDP-43 and Met^85^-TDP-35, EGFP tags were added to either the N-terminus or the C-terminus of these two constructs to visualize ectopic expression in SH-SY5Y cells. EGFP-tagged full-length TDP-43 exhibited a nuclear distribution that colocalized with DAPI labeling, whereas EGFP-tagged Met^85^-TDP-35 localized to the cytoplasm exhibiting three distinct patterns: Type I showed that 60 % of transfected cells had a diffuse Met^85^-TDP-35 localization in both the nucleus and the cytoplasm; Type II showed that 25 % of transfected cells had diffuse Met^85^-TDP-35 localization accompanied with small cytoplasmic aggregates, and Type III showed that 15 % of transfected cells had large Met^85^-TDP-35 aggregates in the cytoplasm but no labeling in the nucleus (Online Resource 1). These results recapitulate previous studies [9, 29]. The Met^85^-TDP-35-positive cytoplasmic aggregates did not label with antibodies to ubiquitin or phosphorylated TDP-43 (409/410) (not shown). Moreover, as observed previously for other TDP-43 species with disrupted NLS [9, 29], Met^85^-TDP-35 recruited full-length TDP-43 from the nucleus into the cytoplasm, where it formed aggregates (Online Resource 1).

### Met^85^-TDP-35 forms cytoplasmic aggregates and is toxic to primary motor neurons

**Fig. 4:**
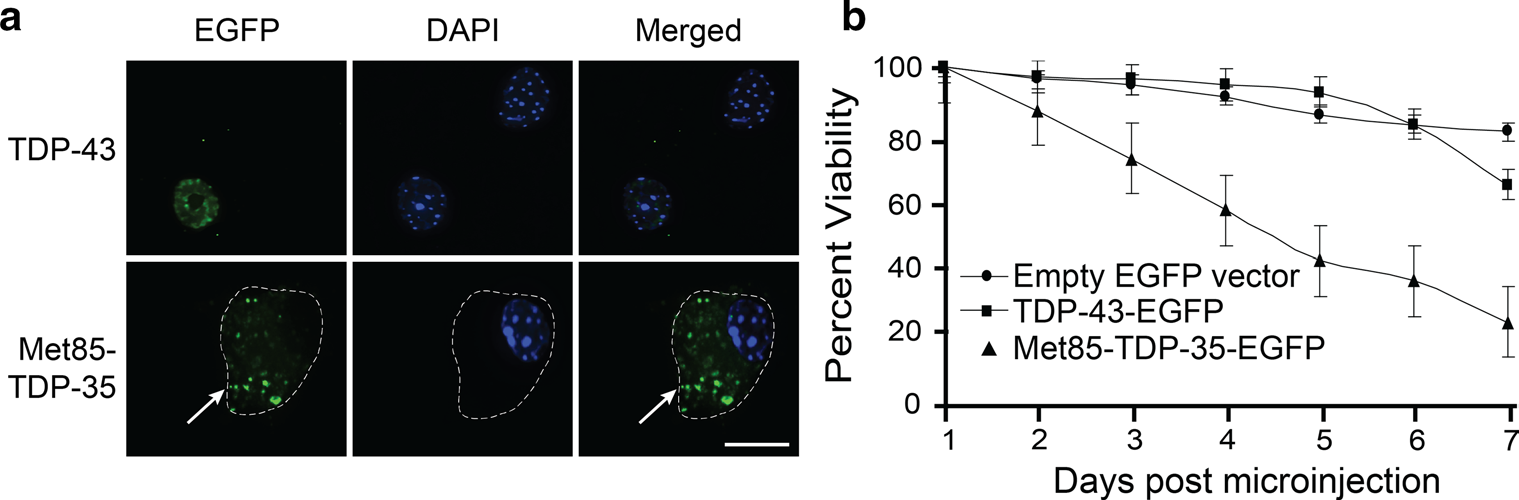
Expression of Met^85^-TDP-35 causes death of primary motor neurons. **a** Expression of EGFP-tagged TDP-43 and EGFP-tagged Met^85^-TDP-35 in primary motor neurons resulted in localization of TDP-43 to the nucleus and Met^85^-TDP-35 to the cytoplasm, where it formed aggregates (*arrow*). **b** A viability curve showed that Met^85^-TDP-35 induced motor neuron death compared to TDP-43 or empty vector alone. *Scale bar* 20 μm

To examine the effect of expressing Met^85^-TDP-35 in an ALS-relevant cell type, plasmids encoding N-terminal EGFP-tagged full-length TDP-43, N-terminal EGFP-tagged Met^85^-TDP-35, or empty vector were expressed in primary motor neurons using intranuclear microinjection. Full-length TDP-43 localized exclusively to the nucleus, whereas Met^85^-TDP-35 was found in the cytoplasm of primary motor neurons, where it appeared as punctate aggregates similar to those observed in transfected SH-SY5Y cells (Fig. 4a). Cell viability assays conducted over a period of 1 week showed that expression of Met^85^-TDP-35 induced motor neuron death with a 50 % loss at day 5 post-microinjection, compared to full-length TDP-43, which exhibited no toxicity with a similar cell viability curve as neurons expressing the empty vector (Fig. 4b).

### Met^85^-TDP-35 expression in ALS

**Fig. 5:**
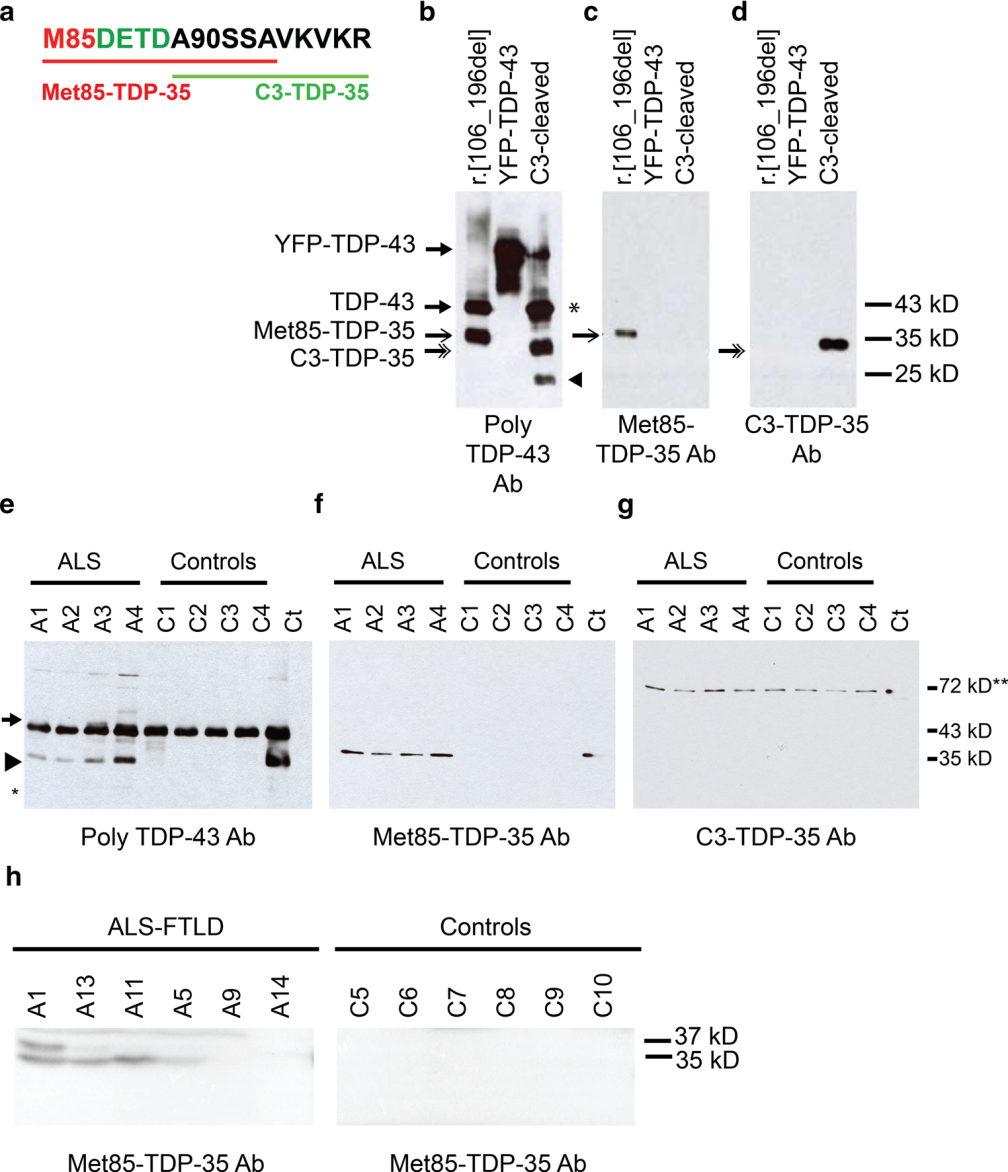
Antibody to Met^85^-TDP-35 detects Met^85^-TDP-35 in ALS and ALS-FTLD tissues. **a** Synthetic peptide sequences used for generation of neo-epitope rabbit polyclonal antibodies specific to Met^85^-TDP-35 (*underlined in red*) and C3-TDP-35 (*underlined in green*). **b**–**d** Neo-epitope antibodies were characterized using lysates of cells expressing TDP-43 r.[106_196del], recombinant YFP-tagged full-length TDP-43 (YFP-TDP-43), and recombinant YFP-tagged full-length TDP-43 cleaved with caspase 3 (C3-cleaved). Note that there is a caspase-3 cleavage site at DEND^13^ of full-length TDP-43, which cleaves the YFP tag (*asterisk*); and at DVMD^19^, generating a cleavage product of 25 kDa (*arrowhead*). **e**–**g** Urea-soluble fractions of spinal cord extracts from four ALS cases (*A1*–*A4*) and four controls (*C1*–*C4*) were immunoblotted with polyclonal TDP-43 antibody (**e**), Met^85^-TDP-35 antibody (**g**), and C3-TDP-35 antibody (**g**). The 35-kDa TDP-43 species (*arrowhead*) in ALS tissue was detected with polyclonal TDP-43 antibody and Met^85^-TDP-35 antibody but not with C3-TDP-35 antibody. Full-length TDP-43 (*arrow*) was not detected by the Met^85^-TDP-35 antibody. The location of the 25-kDa species is shown with an *asterisk*. The 72-kDa band detected by the C3-TDP-35 antibody is non-specific (*double asterisk*). **h** Urea-soluble fractions of prefrontal cortex extracts from six patients with ALS-FTLD and six controls were immunoblotted with Met^85^-TDP-35 antibody. In four out of six patient samples, a 35-kDa species corresponding to Met^85^-TDP-35 was apparent and was absent from all control samples. An additional band of 37 kDa was also apparent in one patient sample, the identity of which is unknown. Cases *A1*, *A4*, *A5*, *A9*, and *A11* also carry *C9orf72* expansions

As our studies in cultured cell lines and primary motor neurons indicated that Met^85^-TDP-35 formed cytoplasmic aggregates, recapitulating a characteristic feature of TDP-43 proteinopathy, we proceeded to assess the pathological relevance of Met^85^-TDP-35 in ALS disease tissues. To study the presence of Met^85^-TDP-35, gray matter from the anterior horn of four sALS and four control lumbar spinal cords were sequentially extracted with buffers of increasing stringency to isolate insoluble proteins (Online Resource 2). Equivalent amounts of low salt, high salt Triton X-100 and urea fractions were immunoblotted with polyclonal TDP-43 antibody (Proteintech), which immunolabeled full-length TDP-43 in all fractions but also a distinct 35-kDa species that was only present in the urea fraction of ALS samples (Online Resource 2). This 35-kDa species was immunolabeled with C-terminal TDP-43 antibody but not with N-terminal TDP-43 antibody (Online Resource 2), confirming that this is a lower molecular weight C-terminal species frequently observed in disease tissues. The 25-kDa species could be observed at much longer exposures of the immunoblots (Online Resource 2). To determine whether this 35-kDa C-terminal species was generated through translation from Met^85^ or from caspase-3 cleavage at DETD^89^, we generated neo-epitope antibodies specific for the extreme N-terminal sequences of these two proteins, termed Met^85^-TDP-35 antibody or C3-TDP-35 antibody, respectively (Fig. 5a). Specificity of the antibodies for their respective protein isoforms was confirmed by immunoblotting using cell lysates expressing TDP-43 r.[106_196del] or full-length recombinant TDP-43 cleaved with caspase-3 in vitro (Fig. 5b–d). Immunoblotting of the urea fractions of ALS and control spinal cords tissues with polyclonal TDP-43 antibody revealed a distinct 35-kDa species present only in ALS samples in addition to the full-length TDP-43 (Fig. 5e). This 35-kDa species was immunolabeled by the neo-epitope Met^85^-TDP-35 antibody (Fig. 5f) but not by C3-TDP-35 antibody (Fig. 5g). Given that the discovery of TDP-43 proteinopathy linked the two previously separate diseases, ALS and FTLD, into one disease spectrum, we examined the presence of Met^85^-TDP-35 in ALS-FTLD brain tissue. Tissues were extracted from the prefrontal cortex, more specifically the most anterior region of Brodmann’s area 10, since this represents a region that usually exhibits extensive TDP-43 proteinopathy in FTLD [5]. A pathological species of 35 kDa was clearly detected in four of the six ALS-FTLD cases, not in controls, and was detected with Met^85^-TDP-35 antibody (Fig. 5h).

**Fig. 6:**
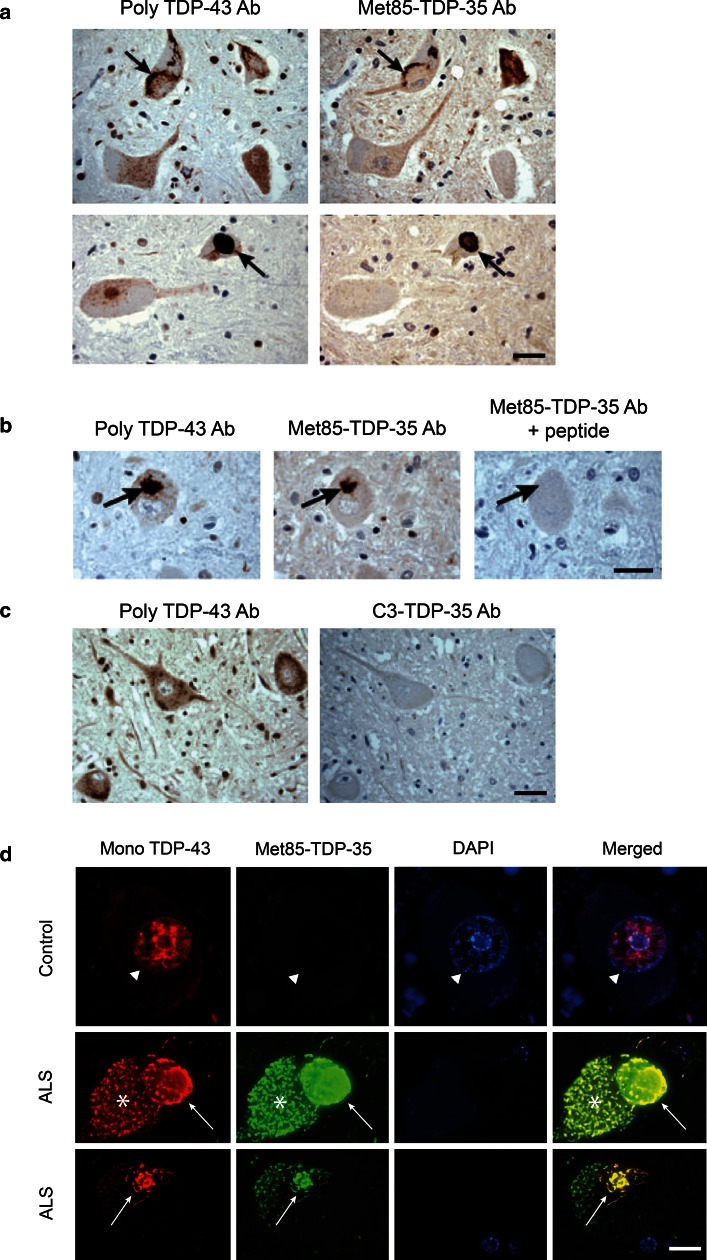
Immunohistochemical characterization of Met^85^-TDP-35 in ALS spinal cord. **a** Immunohistochemical analysis of serial sections from ALS and labeled with polyclonal TDP-43 antibody (poly-TDP-43 Ab) and Met^85^-TDP-35 antibody (Met^85^-TDP-35 Ab). Note that Met^85^-TDP-35 immunoreactivity is localized to TDP-43-positive pathology (*arrows*). **b** Met85-TDP-35 immunoreactivity was ablated by competition with the immunizing peptide (*arrows*). **c** There was no coincident labeling of TDP-43 pathology with C3-TDP-35 antibody (C3-TDP-35 Ab). **d** Double immunofluorescence labeling of control and ALS spinal motor neurons with mouse monoclonal TDP-43 antibody (Mono TDP-43; *red*) and Met^85^-TDP-35 Ab (*green*), nuclei stained with DAPI (*blue*). Note labeling of motor neuron nucleus in control spinal cord with mono TDP-43 antibody but not with Met^85^-TDP-35 antibody (*arrowhead*), indicating lack of cross reactivity of Met85-TDP-35 Ab with full-length TDP-43. Conversely, Met^85^-TDP-35 immunoreactivity co-localized with TDP-43 pathology in motor neurons of ALS cases (*arrows*). *Asterisk* indicates lipofuscin. *Scale bars* 20 μm (**a**–**c**); 5 μm (**d**)

To determine whether Met^85^-TDP-35 is incorporated within pathological inclusions, immunohistochemical labeling with polyclonal TDP-43 antibody and Met^85^-TDP-35 antibody was performed on serial sections of ALS lumbar spinal cord. Met^85^-TDP-35 antibody co-labeled the same pathological inclusions as polyclonal TDP-43 antibody (Fig. 6a), and this labeling could be abrogated by competition with the peptide used to generate the Met^85^-TDP-35 antibody (Fig. 6b). In contrast, there was no labeling of TDP-43-positive inclusions with C3-TDP-35 antibody (Fig. 6c). Double fluorescence labeling showed labeling of the nucleus in normal motor neurons of control cases with mouse monoclonal TDP-43 antibody, and absence of labeling with Met^85^-TDP-35 antibody, confirming that Met^85^-TDP-35 neo-epitope antibody did not cross-react with full-length TDP-43 (Fig. 6d). In contrast, double fluorescence labeling of motor neurons in ALS lumbar spinal cord showed co-labeling of both round and skein like inclusions with the polyclonal TDP-43 antibody and Met^85^-TDP-35 antibody, confirming that Met^85^-TDP-35 is incorporated together with full-length TDP-43 within the pathological inclusions characteristic of ALS (Fig. 6d).

## Discussion

TDP-43 proteinopathy is the hallmark of the majority of ALS-FTLD cases, and biochemical characterization of pathological TDP-43 from disease tissues indicates that abnormal lower molecular weight TDP-43 species comprising the C-terminal region may play a role in disease pathogenesis [16]. Understanding the etiology of such species is important to advancing our understanding of TDP-43 pathogenicity. Here, we have provided evidence that the 35-kDa lower molecular weight species observed in diseases tissues can be derived from expression of an alternative splice variant of TDP-43. This splice variant, TDP-43 r.[106_196del], is upregulated approximately four-fold in ALS spinal cord compared to controls and is generated using non-canonical splice sites that skip the base pairs 106–196 in exon 2 of *TARDBP*, causing a frame shift and generating a premature termination codon 190 bp downstream from the first ATG. However, we have shown that a translation reinitiation event from the third ATG^Met85^ leads to the expression of an N-terminally truncated TDP-43 protein, denoted here as Met^85^-TDP-35, that has a disrupted NLS. Use of ATG^Met85^ as an alternative TDP-43 start site has been reported previously in cell culture studies showing that TDP-43 species of 35 kDa can be generated independent of caspase-3 cleavage [9, 17, 29]. Here, we have established the physiological relevance of this isoform in ALS and FTLD-TDP and shown that it can be generated via an abnormal splicing event.

As has been reported for other N-terminally truncated 35-kDa species of TDP-43 [9, 29], Met^85^-TDP-35 had reduced solubility, a cytoplasmic localization and formed aggregates when expressed in human neuroblastoma cells, all of which are predominant features of pathological TDP-43 in ALS and FTLD [1, 15]. Moreover, co-expression of Met^85^-TDP-35 with full-length TDP-43 resulted in a redistribution of full-length TDP-43 from the nucleus to the cytoplasm, where it was sequestered into Met^85^-TDP-35-positive inclusions. These inclusions were not labeled with ubiquitin or TDP-43 phosphorylation-dependent antibodies, supporting earlier studies showing that aggregation can occur in the absence of these modifications [12, 13, 22]. Given that TDP-43 has been shown to form homodimers [23], Met^85^-TDP-35 could potentially interact with TDP-43 and sequester it into cytoplasmic inclusions, thereby providing a possible mechanism for a gain-of-function toxicity of cytoplasmic Met^85^-TDP-35 and at the same time a loss-of-function resulting from a depletion of nuclear TDP-43. This potential mechanism of action has been proposed by others and our data fully support this premise [9, 26]. Importantly, our findings have shown that expression of Met^85^-TDP-35 in primary motor neurons led not only to formation of cytoplasmic aggregates, but also to decreased cell viability, demonstrating that Met^85^-TDP-35 can induce death of the neuronal type affected in ALS.

Although original studies have reported lower molecular weight TDP-43 species of ~25 kDa in FTLD brain tissues [1, 16], we consistently observed a protein species of 35 KDa as the predominant species on immunoblots of urea-soluble lysates extracted from gray matter of the anterior horn of ALS lumbar spinal cord, with a 25-kDa species observable after longer exposures. This may reflect differences in spinal cord versus brain tissues, or perhaps the extent of degeneration in the regions sampled. In this regard, in all cases used for the current study, areas adjacent to the regions sampled for biochemistry were confirmed to have remaining motor neurons with TDP-43 pathology by immunohistochemical analysis. Indeed, subsequent to the original descriptions of lower molecular weight species of TDP-43 in disease tissues, several other groups have also reported the presence of 35-kDa species in both ALS and FTLD tissues [23, 30]. To determine if the pathologically associated TDP-43 species of 35 kDa observed in ALS spinal cord tissues was derived from alternate translation initiation or caspase-3 cleavage, we generated neo-epitope antibodies specific for the respective N-terminal sequences, starting at Met^85^ or Asp^90^. The 35-kDa species observed on immunoblots of ALS tissues was detected using Met^85^-TDP-35 antibody but not C3-TDP-35 antibody, indicating that in all the samples tested in this study (*n* = 12 ALS cases), this 35-kDa species is generated through an alternate translation event starting at Met^85^. Analysis of urea-soluble fractions from prefrontal cortex also revealed that a 35-kDa lower molecular weight species of TDP-43 was clearly present in four of six ALS-FTLD cases, absent in controls, and was also labeled with Met^85^-TDP-35 antibody, again suggesting that this pathological form of TDP-43 in the brain is generated by alternate translation starting at Met^85^.

Immunohistochemical analysis showed labeling of TDP-43 pathology in motor neurons of ALS spinal cord with Met^85^-TDP-35 antibody, but not with C3-TDP-35 antibody. Interestingly, Met^85^-TDP-35 antibody labeling was specific to TDP-43 pathology in motor neurons, suggesting that the event leading to expression of Met^85^-TDP-35 may be motor neuron specific. This could be important for understanding the selective vulnerability of motor neurons, and possibly other affected neuronal types in ALS-FTLD, especially as expression of Met^85^-TDP-35 can kill motor neurons. Future studies will be aimed at investigating this possibility. One of the normal functions of TDP-43 is in the regulation of alternative splicing and prior studies have shown that downregulation of TDP-43 can lead to changes in the splicing profile of TDP-43 itself [2, 18]. As such, it is possible that the abnormalities of TDP-43 observed in ALS-FTLD could lead to expression of the TDP-43 r.[106_196del] transcript that generates Met^85^-TDP-35. Another possible mechanism is RNA perturbations caused by G_4_C_2_ hexanucleotide repeat expansions in *C9orf72*, the most common known genetic cause of ALS-FTLD and typified by TDP-43 pathology [10, 19, 20]. RNA foci generated by the expansions are observed in disease-affected neurons, sequestering RNA-binding proteins from their normal targets, which could include TDP-43 and thereby lead to splicing irregularities and generation of Met^85^-TDP-35. In support of this, both the TDP-43 r.[106_196del] transcript and Met^85^-TDP-35 were found in the ALS cases with *C9orf72* repeat expansions, as well as sALS cases.

Based on our observations in culture, expression of Met^85^-TDP-35 would lead to sequestering of full-length TDP-43 from the nucleus to form cytoplasmic aggregates and cause neuronal death, an idea that has been promulgated previously [26]. As such, understanding the mechanism that generates Met^85^-TDP-35 could have therapeutic potential. Finally, although we have shown that the splice variant TDP-43 r.[106_196del] can generate Met^85^-TDP-35, there could be additional mechanisms that lead to use of ATG^Met85^ as an alternate translation start site and these remain to be explored.

In conclusion, we have identified a splice variant of TDP-43 that is upregulated in ALS and generates a protein species of 35 kDa through alternate translation initiation at ATG^Met85^, called Met^85^-TDP-35. A neo-epitope antibody specifically recognizing Met^85^-TDP-35 labeled the pathologically associated 35-kDa species on immunoblots of ALS-FTLD tissue extracts, and co-labeled TDP-43 pathology. Presence of Met^85^-TDP-35 was specific to affected motor neurons and could provide a potential mechanism for TDP-43-mediated neurotoxicity in disease.

## Electronic supplementary material

Below is the link to the electronic supplementary material.

Supplementary material 1 (PDF 3471 kb)

Supplementary material 2 (PDF 3690 kb)
