# Supplementary material for: Low molecular weight species of TDP-43 generated by abnormal splicing form inclusions in amyotrophic lateral sclerosis and result in motor neuron death
Source: Acta Neuropathol. 2015 Mar 19;130(1):49–61. doi: 10.1007/s00401-015-1412-5 (PMC4468798; doi:10.1007/s00401-015-1412-5)
Supplement: Supplementary file 1 — Supplementary material 1 (PDF 3471 kb) [file 401_2015_1412_MOESM1_ESM.pdf]

Acta Neuropathologica

**Low molecular weight species of TDP-43 generated by abnormal splicing**

Shangxi Xiao, Teresa Sanelli, Helen Chiang, Yulong Sun, Avijit Chakrabartty, Julia Keith, Ekaterina Rogaeva, Lorne Zinman and Janice Robertson[1]
[1] Corresponding author: jan.robertson@utoronto.ca, Tanz Centre For Research in Neurodegenerative Diseases, Department of Laboratory Medicine and Pathobiology, University of Toronto, Toronto, ON, Canada, M5T 2S8

**Electronic Supplementary Material 1**

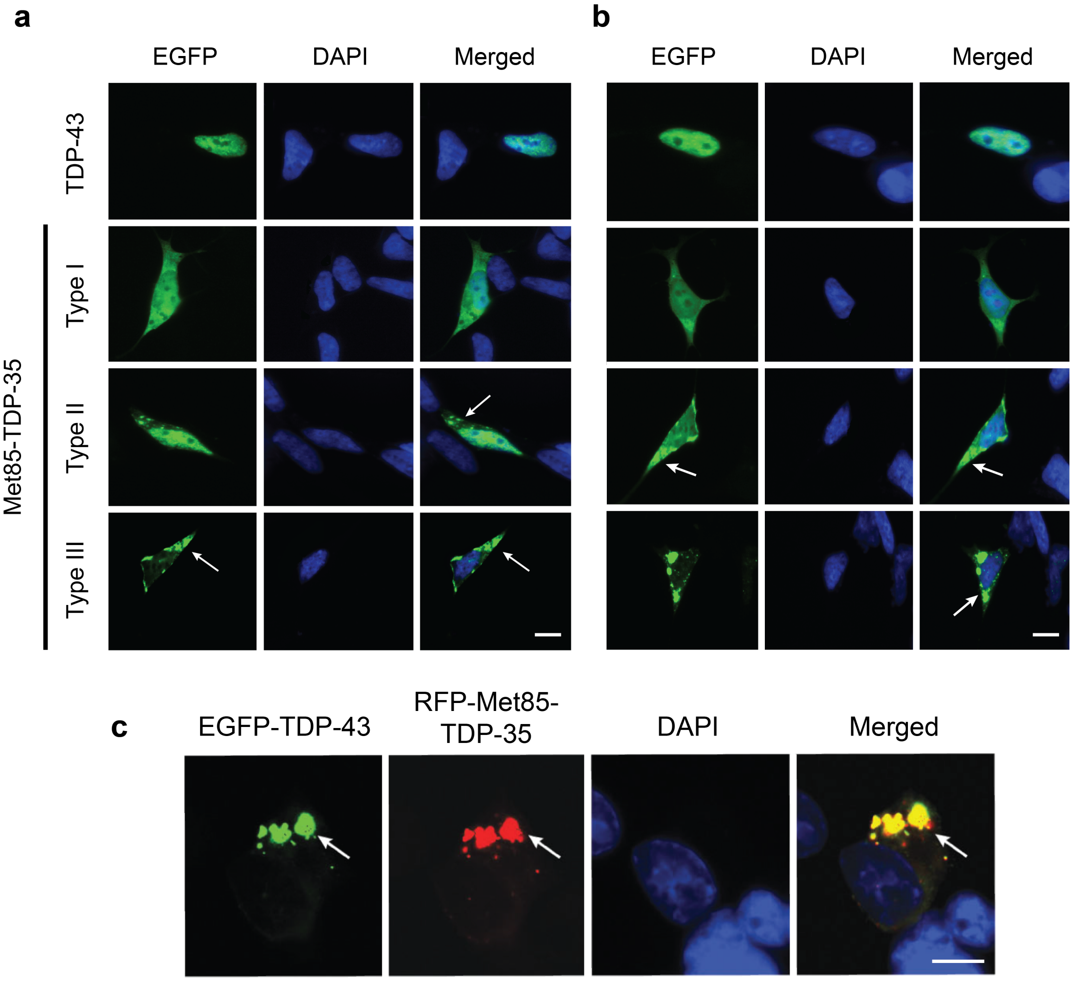


**Fig. S1** Expression pattern of full-length TDP-43 and Met$^{85}$-TDP-35 in transfected SH-SY5Y cells. C-terminally tagged (a) and N-terminally tagged (b) EGFP-TDP-43 and EGFP-Met$^{85}$-TDP-35 demonstrated different subcellular localizations, with TDP-43 in the nucleus and Met$^{85}$-TDP-35 exhibiting three types of expression patterns. In type I, Met$^{85}$-TDP-35 was diffuse throughout the whole cell (including both nucleus and cytoplasm); in type II, Met$^{85}$-TDP-35 was diffuse throughout the whole cell but also formed cytoplasmic aggregates (arrow); and in type III, Met$^{85}$-TDP-35 formed large cytoplasmic aggregates (arrow) and was absent from the nucleus. (c) In SH-SY5Y cells co-expressing EGFP-TDP-43 and RFP-Met$^{85}$-TDP-35, TDP-43 redistributed from the nucleus to the cytoplasm and colocalized to the same inclusion bodies (arrow) as Met$^{85}$-TDP-35. Scale bars = 10 μm
