# Supplementary material for: Low molecular weight species of TDP-43 generated by abnormal splicing form inclusions in amyotrophic lateral sclerosis and result in motor neuron death
Source: Acta Neuropathol. 2015 Mar 19;130(1):49–61. doi: 10.1007/s00401-015-1412-5 (PMC4468798; doi:10.1007/s00401-015-1412-5)
Supplement: Supplementary file 2 — Supplementary material 2 (PDF 3690 kb) [file 401_2015_1412_MOESM2_ESM.pdf]

Acta Neuropathologica

Low molecular weight species of TDP-43 generated by abnormal splicing

Shangxi Xiao, Teresa Sanelli, Helen Chiang, Yulong Sun, Avijit Chakrabartty, Julia Keith, Ekaterina Rogaeva, Lorne Zinman and Janice Robertson[1]
[1] Corresponding author: jan.robertson@utoronto.ca, Tanz Centre For Research in Neurodegenerative Diseases, Department of Laboratory Medicine and Pathobiology, University of Toronto, Toronto, ON, Canada, M5T 2S8

Electronic Supplementary Material 2

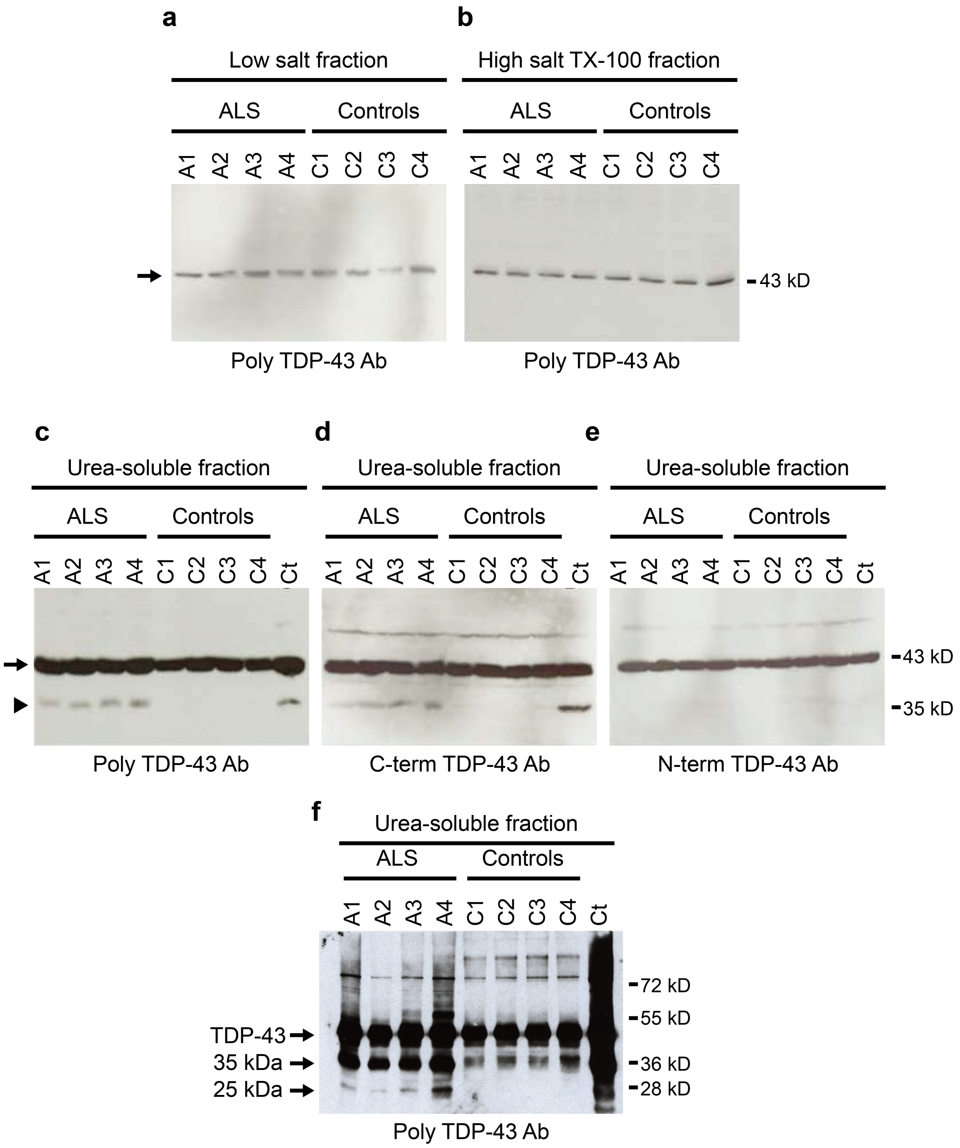

Fig. S2 Biochemical analysis of TDP-43 in spinal cord extracts of ALS and control cases. Immunoblotting equivalent amounts of (a) low salt (b) high salt Triton X-100 and urea-soluble fractions using polyclonal TDP-43 antibody (c) showed that full-length TDP-43 (arrow) was present in all fractions. A distinct 35 kDa species (arrowhead) was noticeable only in the urea-soluble fraction (c) and was present only in ALS samples and not in controls. This 35 kDa species exhibited similar MW as Met^{85}-TDP-35 (Ct = control) and was immunolabeled with C-terminal TDP-43 antibody (d) and not N-terminal TDP-43 antibody (e). The 25 kDa species was apparent only during long exposure (f). Cases A1 and A4 also carry *C9orf72* expansions
